# Characterizing the Impact of Compression Duration and Deformation-Related Loss of Closure Force on Clip-Induced Spinal Cord Injury in Rats

**DOI:** 10.3390/neurolint15040088

**Published:** 2023-11-13

**Authors:** Po-Hsuan Lee, Heng-Juei Hsu, Chih-Hao Tien, Chi-Chen Huang, Chih-Yuan Huang, Hui-Fang Chen, Ming-Long Yeh, Jung-Shun Lee

**Affiliations:** 1 Division of Neurosurgery, Department of Surgery, National Cheng Kung University Hospital, Tainan 701, Taiwan; ftl053@gmail.com (P.-H.L.); nautilus1126@gmail.com (C.-H.T.); charles042085@hotmail.com (C.-C.H.); gordon168999@yahoo.com.tw (C.-Y.H.); 2 Department of Neurosurgery, Tainan Municipal Hospital, Tainan 701, Taiwan; nseastevil@gmail.com; 3 Department of Cell Biology and Anatomy, College of Medicine, National Cheng Kung University, Tainan 701, Taiwan; t96031038@gmail.com; 4 Department of Biomedical Engineering, National Cheng Kung University, Tainan 701, Taiwan; mlyeh@mail.ncku.edu.tw; 5 Medical Device Innovation Center, National Cheng Kung University, Tainan 701, Taiwan; 6 Institute of Basic Medical Sciences, College of Medicine, National Cheng Kung University, Tainan 701, Taiwan

**Keywords:** clip compression, compression duration, clip fatigue, closure force, spinal cord injury

## Abstract

The clip-induced spinal cord injury (SCI) rat model is pivotal in preclinical SCI research. However, the literature exhibits variability in compression duration and limited attention to clip deformation-related loss of closure force. We aimed to investigate the impact of compression duration on SCI severity and the influence of clip deformation on closure force. Rats received T10-level clip-induced SCI with durations of 1, 5, 10, 20, and 30 s, and a separate group underwent T10 transection. Outcomes included functional, histological, electrophysiological assessments, and inflammatory cytokine analysis. A tactile pressure mapping system quantified clip closure force after open–close cycles. Our results showed a positive correlation between compression duration and the severity of functional, histological, and electrophysiological deficits. Remarkably, even a brief 1-s compression caused significant deficits comparable to moderate-to-severe SCI. SSEP waveforms were abolished with durations over 20 s. Decreased clip closure force appeared after five open–close cycles. This study offers critical insights into regulating SCI severity in rat models, aiding researchers. Understanding compression duration and clip fatigue is essential for experiment design and interpretation using the clip-induced SCI model.

## 1. Introduction

Spinal cord injury (SCI) is characterized by damage to any portion of the spinal cord. The damage causes temporary or permanent neurological deficits that distress the patients and their caregivers and impose a huge economic burden. To effectively manage this critical health issue, several animal models with SCI have been developed for investigating the pathophysiological mechanisms and potential therapeutic interventions [1,2,3]. Rats are one of the most widely used species in preclinical studies because they exhibit a similar pathological progression to humans [4]. In rats, varied manners have been used to induce/mimic SCI with different types of causes, such as contusion, distraction, dislocation, transection, exposure to neurotoxic chemicals, and compression [1,5,6]. Compressive models are commonly used to generate persisting compression after an acute spinal cord impact, mimicking SCI caused by fracture dislocations and burst fractures. Among the various methods for inducing compressive damage to the spinal cord, a clip compression model offers the advantages of low cost, accessibility to experimental equipment, and applicability to all regions of the spinal cord to generate ischemia–reperfusion-type SCI with consistent reproducibility and reliability [1,2,7].

The pathogenesis of clip-induced compression SCI involves an aneurysm or a vascular clip that is closed around the region of interest of the spinal cord to cause an acute injury, followed by a sustained compressive injury as the closed clip remains in situ for a certain period. The duration of clip application effectively and differently affects the severities of numerous aspects of SCI [1,2,8]. However, based on the literature reviews, the clipping time ranges from 10 s to 2 min with respect to various aneurysms or vascular clips [3,9,10,11,12,13,14,15,16,17]. Consequently, it is difficult to compare the findings among studies that have adopted different compression durations. A comparative study determining the effects of compression duration on the severity of functional, histological, and electrophysiological deficits in a compression SCI model is warranted. Moreover, the closure force of clips affects the severity of compression-induced SCI [12]. The closure force varies depending on the strength and physical characteristics of the spring attached to the clip. Although the closure force is relatively stable among clips obtained from the same manufacturing resource, few studies have assessed whether repetitive application of the clip would result in clip deformation and reduction of closure force.

In light of the diverse range of clipping durations in previous studies, we have recognized a pressing need for a comprehensive investigation into how the duration of compression, particularly under the specified condition of an 80-g compression force, impacts the severity of compression-induced SCI. To bridge this critical gap in our understanding, our primary objective is to investigate the effects of varying compression durations on the degree of functional, histological, and electrophysiological deficits in rats experiencing compression-induced SCI. This research is aimed at shedding light on the intricate relationship between compression parameters and the resulting neurological outcomes, thereby advancing our knowledge in this field. Furthermore, we also examined the effects of repetitive clip application on its closure force. In the first part, compression SCIs with varied compression times were made at the T10 level in rats. We used the Basso–Beattie–Bresnahan (BBB) scale to rate the locomotor functions of rats. The examination of somatosensory evoked potential (SSEP) and Luxol fast blue (LFB) staining were used to determine the compression-induced electrophysiological and histological deficits, respectively. In the second part, we used a tactile pressure mapping system to determine the closure force of clips that were repetitively used for certain times. Our results provided information for optimizing the protocol for establishing the clip compression SCI model in rats.

## 2. Materials and Methods

### 2.1. Animals and Grouping

**Figure 1:**
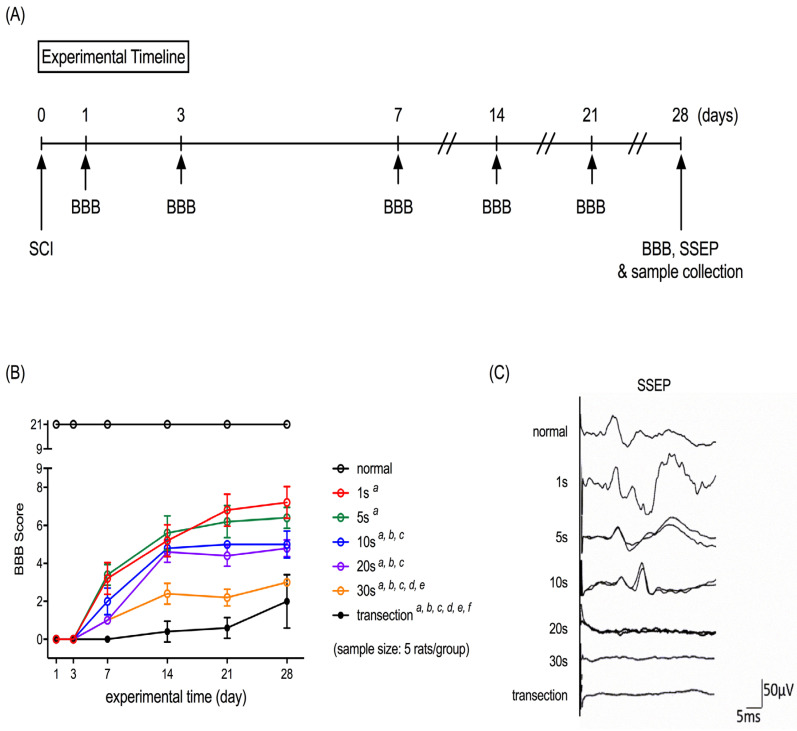
Effects of compression duration on the severity of locomotor functional and electrophysiological deficits in a clip compression rat model of SCI. (**A**) Experimental timeline of this presented study. (**B**) Quantitative results of the Basso–Beattie–Bresnahan scores. (**C**) Representative micrographs of traces of somatosensory evoked potential. *^a^ p* < 0.05, vs. normal, *^b^ p* < 0.05, vs. 1 s, *^c^ p* < 0.05, vs. 5 s, *^d^ p* < 0.05, vs. 10 s, *^e^ p* < 0.05, vs. 20 s, *^f^ p* < 0.05, vs. 30 s.

**Figure 2:**
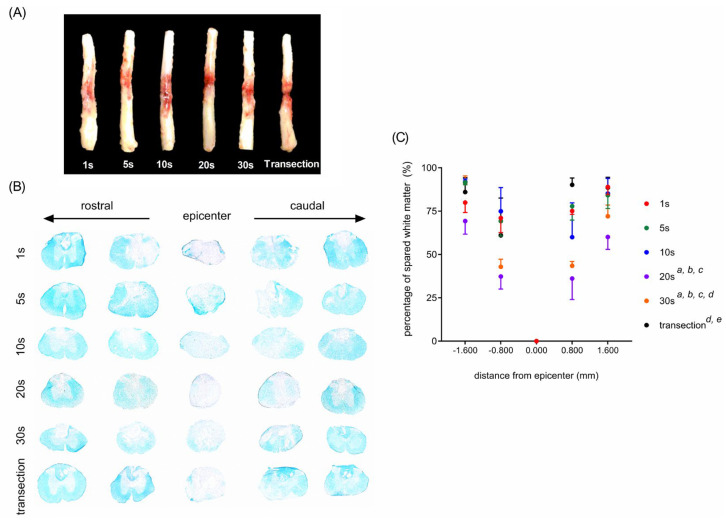
Effects of compression duration on the severity of histological deficits in a clip compression rat model of SCI. (**A**) Representative photographs of dissected spinal cords after clip compression and transection injuries. (**B**) Representative micrographs of LFB staining of coronally sectioned spinal cord sections. (**C**) Quantitative results of LFB staining. *^a^ p* < 0.05, vs. 1 s, *^b^ p* < 0.05, vs. 5 s, ^*c*^ *p* < 0.05, vs. 10 s, *^d^ p* < 0.05, vs. 20 s, *^e^ p* < 0.05, vs. 30 s.

**Figure 3:**
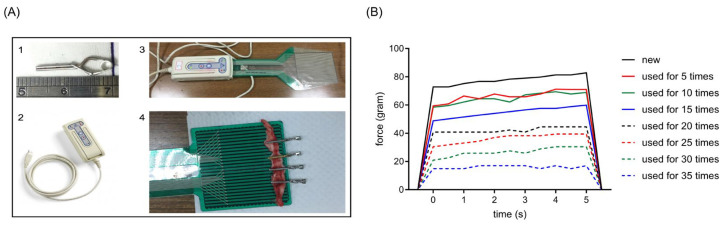
Effects of repetitive clip application on the closure force of the clip. (**A**) Photographs of the versatile I-Scan tactile pressure mapping system. (1) Photograph of aneurysm clips used in this study. (2) Photograph of the data acquisition electronics of the pressure mapping system. (3) Photograph of the data acquisition electronics connected with the plate sensor. (4) Photograph of the experimental setting for detecting the closure force of the clips. (**B**) Quantitative results of the closure force of the clips.

All the animal experiments were conducted as per the U.S. National Institutes of Health Animal Protection Guidelines and with the approval of the National Cheng Kung University (NCKU) Institutional Animal Care and Use Committee. Male Sprague–Dawley rats aged 12 weeks (250–300 g) were purchased from BioLASCO (Nangang, Taipei City, Taiwan) and maintained in the Laboratory Animal Center of NCKU. Each cage housed two rats under an 11-h light/13-h dark cycle (lights on at 7 AM) at a stable temperature (24 ± 1 °C) and humidity in the facility. The rats were given free access to food and water. A total of 71 rats were used in this study. Among them, 35 rats were equally divided into the following seven groups: normal, 1-s, 5-s, 10-s, 20-s, 30-s, and transection groups, and subjected to behavioral, electrophysiological, and histological examinations. The relevant results are shown in Figure 1 and Figure 2. One extra rat was used to evaluate the endurance of the aneurysm clips. Its spinal cord was freshly isolated and replaced on the plate sensor of the pressure mapping system to detect the closure force of the aneurysm clips applied to the spinal cord. The relevant results are shown in Figure 3. The remaining 35 rats were utilized for cytokine arrays, which were conducted on the 7th day following the induction of SCI. 

### 2.2. Induction of SCI

Rats were anesthetized with Zoletil (40 mg/kg, i.p.; Virbac, Carros, France), treated with enrofloxacin (5 mg/kg, i.p.; Bayer AG, Leverkusen, Germany), and placed in a prone position. A dorsal 2-cm longitudinal incision was made over the T9–T11 vertebrae of rats. The spinal process of T10 vertebrae was removed, followed by a T10 laminectomy to expose the underlying spinal cord. An aneurysm clip with 80-g closure force (model: Sugita, Mizuho Logistics, Chiba, Japan) was extradurally applied to close the spinal cord for varied durations, i.e., 1 s, 5 s, 10 s, 20 s, and 30 s. The transection SCI group rats received total transection damage to the T10 spinal cord. Another group of rats that underwent the same surgical procedure without clip compression was termed as the normal group and served as the control group. Then, the wound was closed in layers, and the rats were allowed to recover from anesthesia under a warm blanket. Postoperatively, the rats were housed in one per cage to prevent them from biting each other.

### 2.3. BBB Scoring

Functional outcome assessments of locomotor were conducted using the BBB scale (range: 0–21) on the 1st, 3rd, 7th, 14th, 21st, and 28th days after SCI as described in our previous study [18]. The rats were introduced to an open field and allowed to move freely. The walking pattern of the rat was recorded for 5 min with a digital camera. The locomotor function of the hindlimbs of rats was evaluated using two experimenters blinded to the treatment groups of rats.

### 2.4. SSEP Measurement

Twenty-eight days after SCI, the rats were anesthetized using Zoletil (40 mg/kg, i.p.; Virbac) and positioned on a stereotactic apparatus in a hip-extended and head-flexed posture. SSEPs were recorded using bipolar needle electrodes placed into the C2–C3 interspinous ligament and the nearby subcutaneous tissue. A ground electrode was placed in the shoulder, ipsilateral to the side being stimulated. A pair of stimulating electrodes was inserted into the plantar aspect of the foot at a 2-mm distance. Fifty 200 μs 0.5 mA pulses were delivered at 0.5 Hz, generating an orthodromic stimulation through sensory fibers of the tibial nerve. The recorded signals were averaged from the 50 times repetitive recording at a band-pass filter setting of 50–5000 Hz, with a 20-ms time base.

### 2.5. LFB Staining

LFB staining was used to examine the degree of myelination in the spinal cords of the rats. After the SSEP measurement, the anesthetized rats were transcardially perfused with normal saline, and their spinal cords were quickly removed and post-fixed with 4% paraformaldehyde for 2 days. Serial spinal cord transverse cryosections (20-μM thick, 400-μm intervals) were prepared and rinsed with 100% and 95% ethanol. Following the steps, the sections were then stained with LFB staining solution (0.1% LFB in 95% ethanol with 0.5% acetic acid) at 60 °C, rinsed in 95% ethanol, and differentiated in 0.05% lithium chloride solution.

### 2.6. Cytokine Arrays

The frozen spinal cord specimens were homogenized with iced lysis buffer (Cat. #: 78510, Thermo Fisher Scientific, Waltham, MA, USA) containing protease inhibitors (Cat. #: 04693116001, Roche, Basel, Switzerland) and phosphatase inhibitors (Cat. #: 4906837001, Roche). The homogenates were centrifuged at 15,000× *g* for 10 min at 4 °C, and the supernatants were collected. The expression levels of cytokines in the spinal cord samples were determined using the cytokine antibody array (Cat. #: AAR-CYT-1-2, RayBiotech Life, Peachtree Corners, GA, USA) by following the instructions of the manufacturer.

### 2.7. Measurement of the Closure Force of the Aneurysm Clips

The versatile I-Scan tactile pressure mapping system (Tekscan, Inc., South Boston, MA, USA) was used to measure the closure force of the aneurysm clips. A freshly removed spinal cord tissue was placed on the plate sensor of the I-Scan tactile pressure mapping system, and the clips were applied to it. The readings of the closure force generated from the aneurysm clip were recorded for 5 s and plotted.

### 2.8. Statistical Analyses

Data are expressed as mean ± standard error of the mean. Significance was set at *p* < 0.05. The results of BBB scoring were analyzed with repeated measures of two-way ANOVA followed by Sidak’s multiple comparisons. The results of LFB staining were analyzed with ordinary two-way ANOVA followed by Tukey’s multiple comparisons. The expression levels of selected cytokines in the rat spinal cords were analyzed with one-way ANOVA followed by Tukey’s multiple comparisons.

## 3. Results

### 3.1. Effects of Compression Duration on the Severity of Functional Deficits in a Clip Compression Rat Model of SCI

We induced the compression SCI in rats by applying an aneurysm clip on their spinal cord for 1, 5, 10, 20, and 30 s. The locomotor functions of these rats were evaluated with the BBB scoring scale on the 1st, 3rd, 7th, 14th, 21st, and 28th days after SCI (Figure 1A). Repeated measures of two-way analysis of variance showed that the rats with SCI induced by a 1-, 5-, 10-, 20-, or 30-s compression exhibited a lower BBB score than the controls (Figure 1B). The degree of locomotor impairments was comparable between the rats with SCI induced by 1- and 5-s compression (Figure 1B). As the compression duration increased to >10 s, the severity of locomotor impairment increased. The results of BBB scoring obtained from the 10-s and 20-s groups were similar, and both were lower than those obtained from the 1-s and 5-s compression groups (Figure 1B). As the compression duration reached 30 s, the severity of locomotor impairments in rats was higher than those induced by a 1-, 5-, 10-, and 20-s compression (Figure 1B) but lower than that induced by complete transection (Figure 1B).

### 3.2. Effects of Compression Duration on the Severity of Electrophysiological Deficits in a Clip Compression Rat Model of SCI

Measurement of SSEP was used to determine the somatosensory nerve conduct in the rats with compression SCI induced by varied compression durations. Our results showed that the amplitude of SSEPs reduced as the compression duration increased, and SSEPs failed to be evoked in the 20-s compression, 30-s compression, and transection groups (Figure 1C).

### 3.3. Effects of Compression Duration on the Severity of Histological Deficits in a Clip Compression Rat Model of SCI

We investigated the effects of compression duration on histological properties. Clip compressions with different compressing durations caused obvious lesions in the spinal cord (Figure 2A). LFB staining was used to determine the effects of SCI on the degree of myelination of the spinal cord of rats. An observable demyelination was evident in the epicenter of the injury site in each group, and the degree of myelination in the regions rostral and caudal to the injury site was decreased as the compression duration increased (Figure 2B). Quantitative results showed that the percentages of spared white matter were comparable between the rats with SCI induced by 1-, 5-, and 10-s compressions (Figure 2C). As the compression duration increased to >20 s, the severity of demyelination increased (Figure 2C). In the rats with SCI caused by a 30-s compression, the severity of demyelination was higher than those with SCIs induced by a 1-, 5-, 10-, and 20-s compressions (Figure 2C).

### 3.4. Effects of Compression Duration on the Expression Levels of Inflammation-Related Cytokines in a Clip Compression Rat Model of SCI

Furthermore, we employed an antibody array to assess the impact of varying compression durations on cytokine expression levels in these rats with SCI (Supplementary Materials Figure S1A). Our results showed that the expression levels of most well-known inflammation-related cytokines, including tumor necrosis factor-α, interleukin-1α, interleukin-1β, interleukin-4, interleukin-6, interleukin-10, lipopolysaccharide-induced CXC chemokine, monocyte chemoattractant protein-1, macrophage inflammatory protein-3α, cytokine-induced neutrophil chemoattractant-2, cytokine-induced neutrophil chemoattractant-3, ciliary neurotrophic factor, C-X3-C motif chemokine ligand 1, granulocyte macrophage-colony stimulating factor, interferon-γ, β-nerve growth factor, tissue inhibitor of metalloproteinase-1, and vascular endothelial growth factor, did not exhibit notable differences among the groups (Figure S1B). Only the expression levels of monocyte chemoattractant protein-1 (MCP-1) (Figure S1C,D) and tissue inhibitor of metalloproteinase-1 (TIMP-1) (Figure S1C,E) were increased in the rat spinal cords following SCI. The MCP-1 level was higher in the SCI groups compared to the control group, with no significant differences related to injury severity (Figure S1C,D). Moreover, compression durations exceeding 10 s led to a significant and duration-dependent increase in TIMP-1 expression in the spinal cord (Figure S1C,E). 

### 3.5. Effects of Repetitive Clip Application on the Closure Force of Clip

We used a tactile pressure mapping system (Figure 3A) to detect the closure force of aneurysm clips that underwent an open–close cycle for a certain number of times. Results showed that the closure force of clips decreased as the number of times the clips were used increased. The closure force of the clips used for the first time was around 80 g (Figure 3B). After undergoing the open–close cycle for 5–10 times, 25% of the closure force of clips was lost (Figure 3B). Only about half of the closure force remained in the clips that were used 20 times (Figure 3B).

## 4. Discussion

This study was designed to address two critical issues in establishing the clip compression SCI model in rats. First, we determined the effects of compression duration on the severity of functional, histological, and electrophysiological deficits in a clip compression rat model of SCI. Second, we tested whether clip fatigue occurs due to deformation and repetitive application and further affects its closure force. The results revealed that the severity of compression-induced functional, histological, and electrophysiological deficits was increased as the compression duration increased. A relatively short duration (1 s) of the aneurysm clip compression was sufficient to induce detectable SCI-related functional, electrophysiological, and histological deficits. Electrophysiologically, SSEP was completely abolished once the compressive period was up to 20 s. In addition, the repetitive application-induced clip deformation and reduction of closure force of the clip was evident after five open–close cycles, suggesting that the consistency of clip closure force should be considered in establishing a consistent SCI animal model with a uniform severity of spinal cord compression. The present study provides information for optimizing the protocol for establishing a clip compression rat model of SCI. 

Two mechanisms of injury are implicated in the clip compression model. One is the initial acute impact resulting from the rapid closure of the clip blades. Another one is the continuing compression when the clip remains in the closing position located on the spinal cord. Herein, we determined the effects of the compression duration on the severity of SCI and the acute impact of clip compression by including the 1-s group. We found that the clip compression for 1 s (acute impact) significantly resulted in detectable functional, electrophysiological, and histological deficits that were similar to the characteristics of moderate-to-severe SCI induced by the New York University impactor system [19]. These results showed that an acute compression impact caused by an aneurysm clip exerting around 80-g closure force was sufficient to generate SCI in rats. A further increment of the compressive period of up to 10 s resulted in the injury corresponding to severe SCI induced by the New York University impactor system. It has been reported that the extent of clip compression-induced histologic and functional impairments is strongly correlated with the closing force of the aneurysm clip [12,20]. Therefore, to generate mild-to-moderate SCIs, the aneurysm clips that exert closure force < 80 g should be used. Furthermore, we found that SSEP became undetectable when the compression duration was >20 s, suggesting that compressing the spinal cord with 80-g force for 20 s was enough to completely block the conduction of the somatosensory signals in rats. This piece of information could be a reference for investigating the somatosensory system in the clip compression rat model of SCI.

Both intracranial aneurysm clips and vascular clips are used for inducing compression SCI in rodents. Intracranial aneurysm clips are single-use tools that are designed to permanently occlude the blood flow, resulting in an aneurysm thrombosis. Vascular clips are reusable and designed to temporally occlude the blood flow with less endothelial trauma during vascular anastomoses intraoperatively. Thus, the closure forces vary widely between the intracranial aneurysm clips and vascular clips. The closure forces of intracranial aneurysm clips and vascular clips are usually set at around 70–180 g [21] and 15–30 g [22], respectively. The huge difference in the closure force between these two kinds of clips is one of the reasons causing the varied compression duration among studies [9,10,11,12,13]. Therefore, the type of clips chosen should depend on the severity of the compression SCI desired.

Pathophysiologically, upon the mechanical injury to the spinal cord, the activated pro-inflammatory cytokines and infiltrated macrophages, neutrophils, and other inflammatory cells deteriorate the secondary injury that persists for weeks [23,24]. The secondary injury is thought to be an ongoing process of cellular destruction, ultimately leading to the death of neuronal cells and the loss of axons in areas not directly affected by the initial injury [23,25,26]. One week following a severe traumatic SCI, astrocytes become reactive [27,28], migrating to the epicenter of the lesion and intertwining with one another between 7~14 days post-injury. These events coincide with the secretion of significant components of the extracellular matrix. Approximately two weeks after the injury, hypertrophic astrocytes encircle the core of the lesion and form a dense, mature wall-like structure known as a glial scar [29,30,31]. In the present study, all electrical and histological assays were conducted on the 28th day post-injury, during the phase after scar formation. Our results revealed that as the compression duration exceeded 20 s, the severity of demyelination increased, and somatosensory evoked potentials (SSEPs) failed to be evoked. Scar formation following SCI results in severe and irreversible loss of function. Therefore, our findings not only indicate that spinal cord compression injury exceeding 20 s with an aneurysm clip exerting an 80-g closure force is sufficient to induce extremely severe SCI comparable to spinal cord transection but also suggest that pronounced scar formation may be involved in SCI induced by such injuries. It is worth noting that the upregulation of most proinflammatory cytokines reaches its peak before 72 h after SCI and subsequently decreases. [32]. This phenomenon may be a key factor contributing to our observation of the absence of a significant difference in pro-inflammatory mediators on the 7th day after SCI.

We demonstrated that repetitive clip applications resulted in reduced closure force. Moreover, the reduction of closure force was proportional to the number of times a clip was reused. This was likely due to the nonelastic deformation of the aneurysm clip after repetitive opening, which occurred when the clip deformation exceeds its tensile yield strain and depends on its materials [13,33]. Papadopoulos et al. examined the effects of repeated use on the closure force of titanium alloy Yaşargil clips and pure titanium Spetzler clips and found that the closure force of both Yaşargil and Spetzler clips were weakened by a 10-min sustained maximal opening [34]. However, after 100 open–close cycles, the pure titanium Spetzler clips exhibited no decline in the closure force. However, the closure force of the titanium alloy Yaşargil clips reduced by 12% [34]. Therefore, pure titanium aneurysm clips might be more capable to minimize the effects of clip deformation and reduction of closure force when used for inducing compression SCI.

## 5. Conclusions

In the present study, we characterized the effects of compression duration on the severity of acute clip compression-induced SCI in rats and underscored the importance of the mechanical endurance of the clips. We also addressed the inherent difference between aneurysm and vascular clips. Our results could be a reference for inducing compression SCI at varied severities by using aneurysm clips exhibiting around 80-g closure force. We believe that our results are useful for optimizing the protocol of establishing a clip compression SCI model in rats.

## Data Availability

The data used in our study are available from the authors on reasonable request.

## Supplementary Materials

The following supporting information can be downloaded at https://www.mdpi.com/article/10.3390/neurolint15040088/s1, Figure S1: Effects of compression duration on cytokine levels in the spinal cords of rats with SCI induced by clip compression.
