# Supplementary material for: Characterizing the Impact of Compression Duration and Deformation-Related Loss of Closure Force on Clip-Induced Spinal Cord Injury in Rats
Source: Neurol Int. 2023 Nov 13;15(4):1383–92. doi: 10.3390/neurolint15040088 (PMC10661265; doi:10.3390/neurolint15040088)
Supplement: Supplementary file 1 [file neurolint-15-00088-s001.zip › neurolint-2692236-supplementary.pdf]

Supplementary Materials: Characterizing the Impact of Compression Duration and Clip Deformation on Clip-Induced Spinal Cord Injury in Rats

Po-Hsuan Lee, Heng-Juei Hsu, Chih-Hao Tien, Chi-Chen Huang, Chih-Yuan Huang, Hui-Fang Chen, Ming-Long Yeh and Jung-Shun Lee

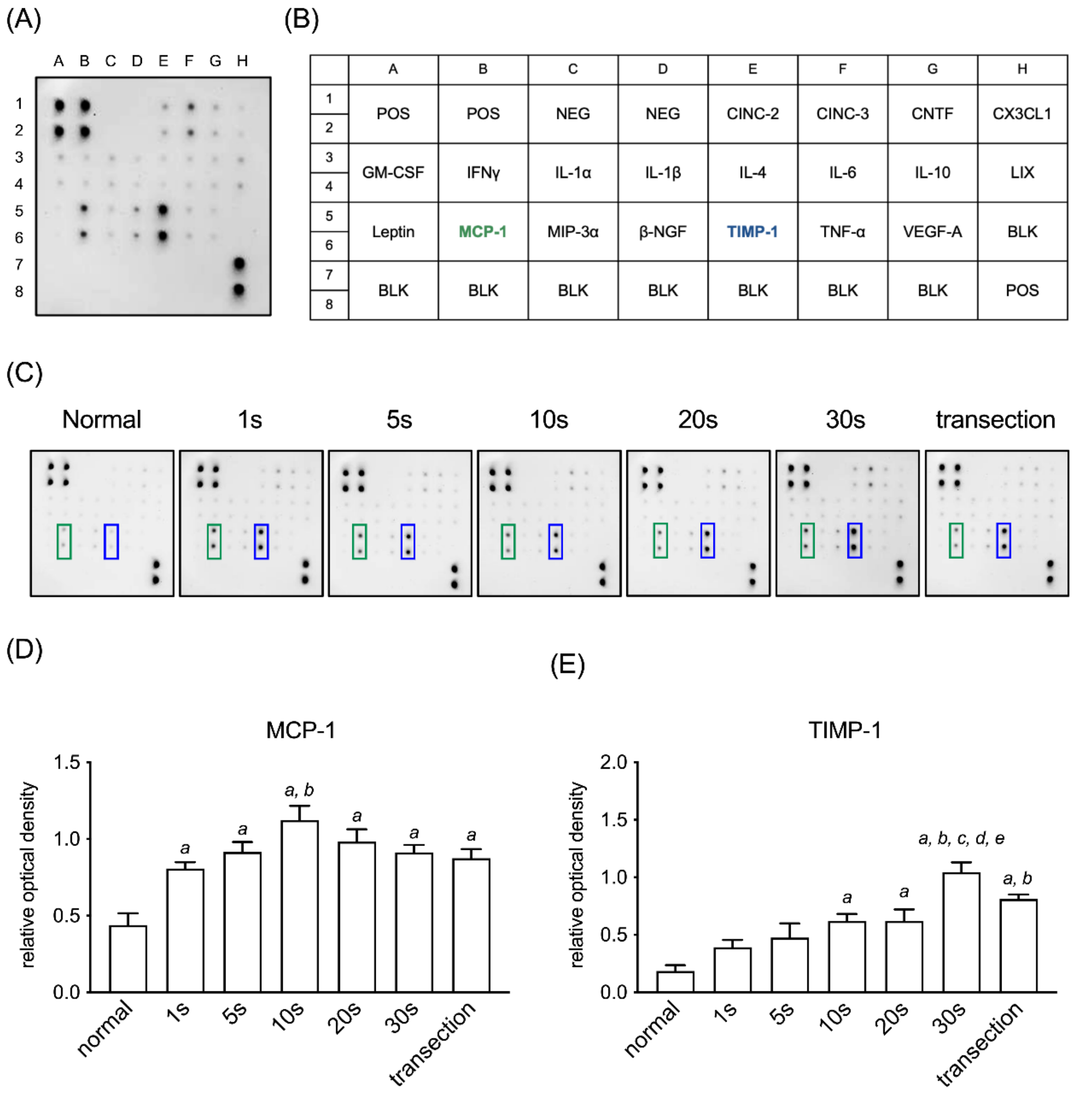

Figure S1. Effects of compression duration on cytokine levels in the spinal cords of rats with SCI induced by clip compression. (A, B) The map of antibody array. POS: positive control; NEG: negtive control; CINC-2: cytokine-induced neutrophil chemoattractant-2; CINC-3: cytokine-induced neutrophil chemoattractant-3; CNTF: ciliary neurotrophic factor; CX3CL1: C-X3-C motif chemokine ligand 1; GM-CSF: granulocyte macrophage-colony stimulating factor; IFNγ: interferon-γ; IL-1α: interleukin-1α; IL-1β: interleukin-1β; IL-4: interleukin-4; IL-6: interleukin-6; IL-10: interleukin-10; LIX: lipopolysaccharide-induced CXC chemokine; MCP-1: monocyte chemoattractant protein-1; MIP-3α: macrophage inflammatory protein-3α; β-NGF: β-nerve growth factor; TIMP-1: tissue inhibitor of metalloproteinase-1; TNF-α: tumor necrosis factor-α; VEGF-A: vascular endothelial growth factor A; BLK: blank. (C) Representative micrographs for antibody arrays. The green and blue frames indicated the signals of MCP-1 and TIMP-1, respectively. (D) Quantitative results of the MCP-1

expression in the spinal cords of rats with SCI induced by clip compression with varying compression durations. (E) Quantitative results of the TIMP-1 expression in the spinal cords of rats with SCI induced by clip compression with varying compression durations. $^{a}p < 0.05$, vs. normal, $^{b}p < 0.05$, vs. 1 sec, $^{c}p < 0.05$, vs. 5 sec, $^{d}p < 0.05$, vs. 10 sec, $^{e}p < 0.05$, vs. 20 sec.
